# Potentially inappropriate medication use in the elderly: physicians’ and hospital pharmacists knowledge, practice, confidence, and barriers

**DOI:** 10.1186/s40780-022-00267-6

**Published:** 2022-12-09

**Authors:** Wuraola Akande-Sholabi, Adeola Fafemi

**Affiliations:** Department of Clinical Pharmacy and Pharmacy Administration, Faculty of Pharmacy, University of Ibadan, Ibadan, Nigeria

**Keywords:** Potential inappropriate medications, Hospital pharmacists, Physicians, Beers criteria, Elderly

## Abstract

**Background:**

Inappropriate medication prescribing and dispensing by physicians and pharmacists is a preventable cause of morbidity and mortality in the elderly. Signifying the importance of healthcare professionals’ knowledge of potentially inappropriate prescribing tools/ guidelines which would improve overall health outcomes. Beers Criteria is one of the most used guidelines that list specific potentially inappropriate medications (PIMs). This study aimed to investigate physicians’ and hospital pharmacists’ awareness, knowledge, practice, confidence, and barriers to the use of Beers criteria.

**Methods:**

A cross-sectional survey was carried out among 66 hospital pharmacists and 31 family medicine physicians working in a teaching hospital in Nigeria, between May and September 2021 using a self-administered questionnaire. Knowledge of PIMs was assessed using 11 clinical vignettes based on the 2019 Beers Criteria. Practice behavior towards elderly patients was assessed using a 5-item statement with a 5-point Likert scale. Perceived barriers to the appropriate use of Beers Criteria were assessed using a 15-item statement. Descriptive and inferential statistics were used to analyze the data.

**Results:**

A total of 66 hospital pharmacists and 31 family medicine physicians participated in the study. Only (24; 36.4%) pharmacists and (4; 12.9%) physicians knew guidelines that listed specific PIMs. In all (21; 31.8%) pharmacists and (11; 35.4%) physicians demonstrated good knowledge of Beers Criteria, while most pharmacists (55; 83.3%) and physicians (29; 93.5%) reported good practice when dealing with elderly patients in terms of asking relevant questions and considering their ages while dispensing medications. Knowledge scores were not significantly associated with hospital pharmacists’ socio-demographics. Most of the physicians and pharmacists were confident in the care provided for geriatric patients. The majority (66; 100.0%) pharmacists and (28; 93.5%) physicians suggested training through seminar presentations as a measure to improve the use of Beers’ criteria. Lack of time to counsel patients was a major barrier recounted by both physicians and pharmacists.

**Conclusion:**

Healthcare professionals that participated in this study had satisfactory practice, although a gap in the knowledge of hospital pharmacists and family medicine physicians was noted. In addition to creating awareness among healthcare professionals and training on PIMs and Beers’ criteria, a multidisciplinary approach to reduce potentially inappropriate prescribing and dispensing would greatly help reduce the incidence of potentially inappropriate medication use among elderly patients.

## Background

Globally, the population of elderly patients (aged 65 and above) is growing and the elderly are more likely to experience age-related changes in pharmacokinetics and pharmacodynamics of the drugs, they are given to treat their multiple pathological conditions [1, 2]. Optimization of drug prescribing in older populations is a priority due to the significant clinical and economic costs of drug-related illnesses. Inappropriate prescribing in the elderly is associated with increases in morbidity, adverse drug events, hospitalization, and mortality [3, 4]. Studies carried out in Nigeria have reported that the use of potentially inappropriate medication among geriatric patients was high [5–8]. Potentially inappropriate medication (PIMs) use is a significant contributor to increased healthcare costs, morbidity, and mortality in the elderly population.

Polypharmacy is associated with adverse outcomes including mortality, falls, drug–drug interactions, adverse drug reactions, increased length of stay in the hospital, and readmission to a hospital soon after discharge. Although, it can be unavoidable in elderly patients its negative effects can be reduced by an inter-professional review of the patient’s case and medications, and the use of well-established tools (explicit and implicit criteria) that help identify potentially inappropriate prescribing, monitor the patient’s active medication use and deprescribing any unnecessary medications can be used to decrease polypharmacy and improve patient-related outcomes [9–11]. Several of such explicit criteria have been developed to help healthcare professionals reduce inappropriate prescribing but the most cited tools are Beers’ criteria (AGS, 2019) [12] and the screening tool of older persons’ prescriptions or the screening tools to alert doctors to right treatment (STOPP/START) criteria [13]. Beers’ criteria and STOPP criteria address potentially inappropriate medications (PIMs), while START criteria address potential prescribing omissions (PPO). The awareness and knowledge of healthcare professionals about potentially inappropriate prescribing are the key elements in preventing the negative outcomes associated with potentially inappropriate prescribing and improving their healthcare outcomes [14].

Inappropriate medication prescribing by physicians is an important preventable cause of morbidity and mortality in the elderly [15]. The awareness and knowledge of primary care physicians in the United States of America on potentially inappropriate prescribing and prescribing guidelines such as Beers’ criteria, STOPP and START criteria were unsatisfactory [15]. A study carried out by Fadare et al.*,* 2013 in Federal Medical Centre, Ido-Ekiti, South-West Nigeria, evidenced that the prescription of potentially inappropriate medications constitutes a major problem, especially when dealing with the elderly [5]. To reduce the prescription of potentially inappropriate medications, there is a need for physicians to be educated and aware of potentially inappropriate prescribing and the different criteria to reduce it. An interdisciplinary approach between healthcare professionals including physicians and pharmacists when implemented is a useful approach in the improvement of prescribing appropriateness in high-risk elderly populations [16].

Pharmacists are essential members of the healthcare system and could play an important role in reducing the occurrence of potentially inappropriate prescribing and potentially inappropriate medication use through medication reconciliation and application of potentially inappropriate prescribing criteria like Beers’ criteria etc. [10].

Pharmacist-led patient counseling and medication reviews have been associated with improved health-related outcomes, medication appropriateness, quality of life, drug knowledge and satisfaction, and reduced healthcare cost and drug-related morbidity and mortality [10, 11]. An interdisciplinary approach to medication review by pharmacists was highlighted as a way of improving physician prescribing practices among the elderly [5]. Prescription monitoring by pharmacists has led to the amendment of prescriptions that are inappropriate, unclear or inadequate showing the importance of pharmacists in an interdisciplinary team to prevent potentially inappropriate prescribing.

Various studies have shown that potentially inappropriate prescribing is a major problem in healthcare [5–8] but there is still a dearth of information on knowledge, practice, and awareness of criteria for potentially inappropriate prescribing in the elderly in Nigeria.

This study aimed to assess knowledge, awareness, confidence, and practice of potentially inappropriate prescribing and dispensing among physicians and hospital pharmacists in a tertiary teaching hospital in Nigeria. This will be an important first step to revealing what can be done to promote better knowledge and practice among hospital pharmacists and physicians on potentially inappropriate medications.

## Methods

### Study design

This study was a questionnaire-guided cross-sectional survey among hospital pharmacists and family medicine physicians working in a teaching hospital between May and September 2021.

### Study setting

This study was carried out at the family medicine unit in University College Hospital Ibadan and the various satellite pharmacy units in University College Hospital Ibadan, Oyo State. University College Hospital is a tertiary health care institution in Nigeria that was established in 1957, it has 850-bed spaces with an occupancy rate of 55-60%. It is a major site for undergraduate and post-graduate residency training for physicians, as well as clinical training for other categories of healthcare practitioners including pharmacists, nurses, and other auxiliary healthcare personnel.

### Study population

Pharmacists working in various pharmacy units and Physicians including consultants, registrars, and medical officers working in the family medicine unit at the University College Hospital, Ibadan during the study period. The minimum degree required to become a pharmacist in Nigeria is a Bachelor of Pharmacy (BPharm). Currently, there are plans to make the Doctor of Pharmacy (PharmD), the minimum entry requirement to practice as a pharmacist in Nigeria. However, only the University of Benin which is situated in Edo State was accredited to award the PharmD degree, although there are existing plans to accredit the other pharmacy institutions for this award.

### Inclusion and exclusion criteria

Eligible participants were registered hospital pharmacists and family medicine physicians, who gave voluntary informed consent to participate in the study. Participating pharmacists and family medicine physicians must have had a minimum of 1 year of practice experience in the hospital pharmacy. Pharmacy students, interns, NYSC pharmacists, and hospital pharmacists, family medicine physicians who were absent from their duty posts and those who declined participation were excluded.

### Sample size determination

This was calculated from the population of hospital pharmacists obtained from the pharmacist’s department in UCH, as an estimate of 80 pharmacists. Based on an estimated population of hospital pharmacists and using an assumption of a 95% confidence interval and a 5% margin of error, a sample size of 66 pharmacists was obtained using Yamane’s sample size determination formula [17]. Total sampling was embarked on; however, some pharmacists did not consent and were excluded from the study. From the sampling frame of 80 pharmacists, five (5) were part of the pretest, four (4) were on annual leave and five (5) did not consent to the study and were therefore excluded from the study, giving a total sample size of 66. Family medicine physicians were calculated from the population of family medicine physicians which was obtained from the family medicine physicians department in UCH as an estimate of 34 physicians. Based on an estimated population of physicians and using an assumption of a 95% confidence interval and a 5% margin of error, a minimum sample size of 31 physicians was obtained using Yamane’s sample size determination formula [17].

### Data collection instruments

The study questionnaire was developed following a literature review of relevant studies and was modified to suit the local context where needed [10, 15, 18]. A questionnaire was the data collection tool for the participants both physicians and pharmacists. The questionnaire used in this study consisted of seven sections: Section 1 obtained information on the respondents’ social-demographic characteristics, years of experience, educational information, and other qualifications. Section 2 assessed the respondents’ confidence and counseling practices concerning older adults. Section 3 evaluated respondents’ practice of Beers’ criteria for potentially inappropriate medication use in the elderly. Section 4 investigated the awareness of respondents on the different criteria for potentially inappropriate prescribing in the elderly. Section 5 assessed respondents’ knowledge of Beers Criteria for potentially inappropriate medication use in the elderly. Knowledge of PIMs listed in Beers’ Criteria was assessed using 11 clinical vignettes like that previously employed by [15, 18] which was updated according to the 2019 version of Beers Criteria (the latest version of the criteria at the time of the study). Section 6 assessed barriers to respondents’ practice of Beers’ Criteria for potentially inappropriate medication use in the elderly, such as lack of time to counsel each patient, lack of knowledge of Beers Criteria, excess workload e.t.c. Section 7 assessed the perception of respondents on ways to improve their use of Beers’ criteria for potentially inappropriate medication use in the elderly using 6 items statement with a 5-point Likert scale ranging from 1 = “Strongly Disagree” to 5 = “Strongly Agree”. A pilot study was conducted with 8 participants from the sample population, where internal consistency was found to be acceptable as demonstrated by a Cronbach alpha value of 0.74.

### Pretest and content validation

Content validity of the questionnaires was carried out by a pharmacist in academia. Appropriate modifications to some questions were made, and some were rephrased to avoid ambiguity. Subsequently, the questionnaire was given to five (5) hospital pharmacists in the general outpatient department of the pharmacy unit and three (3) physicians of the family medicine department in the University College Hospital, Ibadan, Oyo State. Appropriate modifications were made based on the feedback from these respondents and some ambiguous questions were rephrased. These respondents were excluded from the study.

### Sampling and data collection procedure

A consecutive sampling technique/approach was used for participants’ enrolment. Eligible hospital pharmacists and family medicine physicians were approached by visiting individual pharmacists in their respective practice units. Objectives of the study were explained to every participant after which voluntary informed consent was obtained to signify intention to participate in the study. The questionnaire was self-administered by all consented pharmacists and physicians and retrieved within 15–20 min of completion of the questionnaire. Anonymity and confidentiality of response were assured, while participation was entirely voluntary.

### Data analysis

Data entering, cleansing, and analysis were done using IBM Statistical Package for Social Science SPSS (version 23). Descriptive statistics including frequency and percentage were used to describe the socio-demographic characteristics of the respondents. Pearson Chi-square (χ^2^) statistics were used to assess the association between categorical variables such as demographic characteristics, years of experience, respondents’ awareness of criteria for potentially inappropriate prescribing, and respondents’ practice with respondents’ knowledge of Beers’ criteria. The level of significance was set at *P* < 0.05.

For the knowledge questions, a correct answer was assigned “one” and an incorrect answer was given “zero”. Of the 11 knowledge questions, a score of at least nine out of the eleven maximum obtainable score, (i.e. ≥ 80%) was categorized as “good” knowledge, while score < 9 (i.e. < 80%) was categorised as “poor” knowledge. Therefore, a raw score of 9 out of 11 questions that evaluated the knowledge of pharmacists and physicians on Beers Criteria for potentially inappropriate medications indicated good knowledge of respondents. For the 5-item statements on practice with a 5-point Likert scale response, the questions were evaluated on a 5-point Likert rating scale from strongly agree (5) to strongly disagree (1) to explore and evaluate the hospital pharmacists’ and physicians’ practice to reduce potentially inappropriate medication use.

For the 5-item statements on practice with a 5-point Likert scale response, a total score of at least 20 (≥80%) out of the maximum obtainable score of 25 was categorized as ‘good practice’, while a practice score of < 20 was categorized as ‘poor practice’. The binary categorization was adapted from Bloom’s cut-off criteria and other similar studies [19, 20].

## Results

### Socio-demographic characteristics of respondents

There were 34 copies of the questionnaire administered to the physicians and 70 copies of the questionnaire administered to the pharmacists, out of which 31 and 66 were filled completely and returned, giving a response rate of 91.2 and 94.2% respectively. More than half (54.6%) were female, and (46; 47.4%) were aged 30 years and above. About two-thirds (63; 64.9%) of the respondents had a Bachelor of Pharmacy degree. About one-third (24; 24.7%) of the respondents have an MBBS (Bachelor of Surgery, Bachelor of Medicine) degree. See Table 1.

**Table 1:** Socio-demographic characteristics of respondents

Variables	Hospital Pharmacists Frequency (%) (*n* = 66)	Physicians Frequency (%) (*n* = 31)
Gender		
Male	22 (33.3)	22 (71.0)
Female	44 (66.7)	9 (29.0)
Age (years)		
20-29	17 (25.8)	7 (22.6)
30-39	30 (45.5)	20 (64.4)
40-49	16 (24.2)	4 (12.9)
50-59	3 (4.5)	0 (0)
60 and above	0 (0)	0 (0)
Qualifications		
B. Pharm	63 (95.5)	0 (0)
Pharm D	3 (4.5)	0 (0)
MBBS	0 (0)	31 (100.0)
Additional Qualifications		
Masters	4 (6.1)	0 (0)
FWACP	0 (0)	3 (9.6)
MPH	4 (6.1)	2 (6.5)
MWACP	0 (0)	2 (6.5)
M Pharm	1 (1.5)	0 (0)
WAPCP	21 (31.8)	0 (0)
Rank in Institution		
Consultant		3 (9.7)
Senior Registrar		6 (19.3)
Registrar		6 (19.4)
Medical Officer		16 (51.6)
Years of practice		
Less than one	3 (4.5)	8 (25.8)
1-5	26 (39.4)	16 (51.6)
6-10	19 (28.8)	3 (9.7)
11-15	13 (19.7)	4 (12.9)
More than 15 years	5 (7.6)	0 (0)

Key: *B. Pharm* Bachelor of Pharmacy, *Pharm D* Doctor of Pharmacy, *MPharm* Master of Pharmacy, *WAPCP* West African Postgraduate College of Pharmacy, *MBBS* Bachelor of Surgery, Bachelor of Medicine, *FWACP* Fellow of West African College of Physicians, *MPH* Master of Public Health, *MWACP* Member, West African College of Physician

### Respondents’ awareness of the different criteria for potentially inappropriate prescribing in elderly

Table 2 shows that (41; 62.1%) of the hospital pharmacists and (23; 74.2%) of the physicians have not had any previous training or knowledge on potentially inappropriate prescribing and medication use in elderly patients while more than one-third (24; 36.4%) of the hospital pharmacists and (4; 12.9%) of the physicians knew of the criteria used for potentially inappropriate prescribing with Beers’ criteria as the most cited and used.

**Table 2:** Respondents’ awareness of the different criteria for potentially inappropriate prescribing in elderly

Variables	Hospital Pharmacists Frequency (%) (*n* = 66)	Physicians Frequency (%) (*n* = 31)
Have you had any previous training or knowledge on potential inappropriate prescription and medication use for elderly patients and its reduction using any of the criteria (e.g. Beers’ criteria)?		
Yes	25 (37.9)	8 (25.8)
No	41 (62.1)	23 (74.2)
Do you know Any Criteria Used to reduce Inappropriate prescribing?		
Yes	24 (36.4)	4 (12.9)
No	42 (63.6)	27 (87.1)
Please indicate below how often you use the criteria for Potentially Inappropriate Medication Use in Older Patients.		
**Beers’ Criteria**		
Often use	1 (1.5)	0 (0.0)
Sometimes use	8 (12.1)	4 (12.9)
Rarely use	5 (7.6)	0 (0.0)
Know of but never use	24 (36.4)	0 (0.0)
Never heard of resource	28 (42.4)	27 (87.1)
**STOPP and START**		
Often use	1 (1.5)	0 (0.0)
Sometimes use	0 (0.0)	0 (0.0)
Rarely use	5 (7.6)	0 (0.0)
Know of but never use	25 (37.9)	0 (0.0)
Never heard of resource	35 (53.0)	31 (100.0)

### Respondents practice to reduce potentially inappropriate medication use in the elderly

As shown in Table 3 the practice of hospital pharmacists to reduce potentially inappropriate medication use among elderly patients. More than half (34; 51.2%) of the hospital pharmacists ask patients for their ages and the majority (60; 90.9%) ask elderly patients for their medical history. Hospital pharmacists (42; 63.6%) reported that patient age is a key factor when dispensing medications to patients. A majority (64; 97.0%) of hospital pharmacists look for drug interactions when recommending medications and have confidence in the care they provide to the elderly (65; 98.5%). Most hospital pharmacists have satisfactory practice to reduce potentially inappropriate prescribing in the elderly (55; 83.3%). On the other hand, the practice of family medicine physicians to reduce potentially inappropriate prescribing shows (30; 96.7%) ask patients for their ages and medical history, and (27; 87.1%) reported that patient age is a key factor when prescribing medications to patients, twenty-seven (27; 87.0%) look for drug interactions when recommending medications and (28; 90.3%) have confidence in the care they provide to elderly. Only (2; 6.5%) respondents had unsatisfactory practice among family medicine physicians. See Table 4.

**Table 3:** Hospital pharmacists practice to reduce potentially inappropriate medication use in elderly (n = 66)

Variables	Strongly disagree *n* (%)	Disagree *n* (%)	Neutral *n* (%)	Agree *n* (%)	Strongly Agree *n* (%)
I always ask elderly patients for their ages.	4 (6.1)	2 (3.0)	26 (39.4)	24 (36.4)	10 (15.2)
I always ask patients especially elderly patients for their medical history.	2 (3.0)	0 (0.0)	4 (6.1)	33 (50.0)	27 (40.9)
Patients’ age is always a key factor for me when dispensing medications, especially elderly patients.	1 (1.5)	2 (3.0)	11 (16.7)	36 (54.5)	16 (24.2)
I always look for drug interactions especially drug-disease interactions when recommending and dispensing medications to elderly patients.	1 (1.5)	1 (1.5)	0 (0.0)	32 (48.5)	32 (48.5)
I have confidence in the care I provide for elderly patients.	1 (1.5)	0 (0.0)	0 (0.0)	34 (51.5)	31 (47.0)
Practice of Beers’ Criteria					
Poor Practice (< 80%)		11			16.7
Good Practice (> 80%)		55			83.3

Maximum obtainable score = 25; % individual score = score obtained by an individual ÷ by total obtainable score × 100. Strongly agree (SA) = 5, Agree (A) = 4, Undecided (U) = 3, Disagree(D) = 2, Strongly disagree (SD) = 1

**Table 4:** Family medicine physicians’ practice to reduce potentially inappropriate medication use in elderly (*n* = 31)

Variables	Strongly disagree *n* (%)	Disagree *n* (%)	Neutral *n* (%)	Agree *n* (%)	Strongly Agree *n* (%)
I always ask elderly patients for their ages.	0 (0.0)	0 (0.0)	1 (3.2)	7 (22.6)	23 (74.2)
I always ask patients especially elderly patients for their medical history.	0 (0.0)	0 (0.0)	1 (3.2)	5 (16.1)	25 (80.6)
Patients’ age is always a key factor for me when prescribing medications, especially elderly patients.	0 (0.0)	0 (0.0)	4 (12.9)	7 (22.6)	20 (64.5)
I always look for drug interactions especially drug-disease interactions when recommending and dispensing medications to elderly patients.	0 (0.0)	0 (0.0)	4 (12.9)	13 (41.9)	14 (45.2)
I have confidence in the care I provide for elderly patients.	0 (0.0)	0 (0.0)	3 (9.7)	13 (41.9)	15 (48.4)
Practice of Beers’ Criteria					
Poor Practice (< 80%)			2		6.5
Good Practice (> 80%)			29		93.5

Maximum obtainable score = 25; % individual score = score obtained by an individual ÷ by total obtainable score × 100. Strongly agree (SA) = 5, Agree (A) = 4, Undecided (U) = 3, Disagree(D) = 2, Strongly disagree (SD) = 1

### Respondents’ knowledge of Beers’ criteria for potentially inappropriate prescribing in elderly

Respondents’ responses showed that a high percentage of hospital pharmacists had poor knowledge, scoring below 80% (45; 68.2%) of Beers’ Criteria. See Table 5. Although, (56; 84.8%) respondents knew that Corticosteroids plus NSAIDs should be avoided because it increases the risk of peptic ulcer disease or Gastro-Intestinal bleeding, while (54; 84.8%) knew that Benzodiazepines can increase the risk of falls and fractures in elderly patients.

**Table 5:** Hospital pharmacists’ knowledge of Beers’ criteria for potentially inappropriate prescribing in elderly (*n* = 66)

Variables	Response Categories	Frequency (%)
Metoclopramide should be avoided unless for gastroparesis with a duration not exceeding 12 weeks except in rare cases because it can cause extrapyramidal side-effects Correct answer: Yes	Yes	39 (59.1)
	No	10 (15.2)
	I don’t Know	17 (25.8)
Proton-pump Inhibitors should be used for a maximum of 8 weeks in elderly patients except for high-risk patients, erosive oesophagitis, and pathological hypersecretory conditions. Correct answer: Yes	Yes	53 (80.3)
	No	7 (10.6)
	I don’t Know	6 (9.1)
Sertraline would increase the risk of delirium. Correct answer: Yes	Yes	42 (63.6)
	No	5 (7.6)
	I don’t Know	19 (28.8)
SSRIs can increase the risk of falls and should be avoided unless safer alternatives are not available. Correct answer: Yes	Yes	48 (72.7)
	No	3 (4.5)
	I don’t Know	15 (22.7)
Benzodiazepines can increase the risk of falls and fractures in elderly patients. Correct answer: Yes	Yes	54 (81.8)
	No	7 (10.6)
	I don’t Know	5 (7.6)
First Generation antihistamines (Chlorpheniramine) can increase the risk of anticholinergic effects in elderly patients. Correct answer: Yes	Yes	40 (60.6)
	No	8 (12.1)
	I don’t Know	18 (27.3)
Corticosteroids plus NSAIDs should be avoided because it increases the risk of peptic ulcer disease or Gastro-Intestinal bleeding. Correct answer: Yes	Yes	56 (84.8)
	No	2 (3.0)
	I don’t Know	8 (12.1)
Aspirin 325 mg daily may exacerbate existing ulcers or cause new additional ulcers. Correct answer: Yes	Yes	54 (81.8)
	No	9 (13.6)
	I don’t Know	3 (4.5)
Long-acting sulfonylurea (glimepiride, glyburide) should be avoided due to their risk of prolonged hypoglycemia. Correct answer: Yes	Yes	46 (69.7)
	No	9 (13.6)
	I don’t Know	11 (16.7)
A 72-year-old patient has type II diabetes mellitus. The patient is newly diagnosed with hypertension. The patient has experienced recurrent events of hypoglycemia. The patient has no other medical illnesses. Which of the following medications would you consider to AVOID? Correct answer: hydrochlorothiazide.	Metoprolol	25 (37.9)
	Amlodipine	3 (4.5)
	Hydrochlorothiazide	28 (42.4)
	Losartan	4 (6.1)
	I don’t know	6 (9.1)
A 76-year-old patient is newly diagnosed with type II diabetes mellitus. The patient has no other medical illness. You are going to start an oral antidiabetic agent for this patient. Which of the following medications would you consider to AVOID? Correct answer: Glibenclamide	Rosiglitazone	40 (60.6)
	Glibenclamide	13 (19.7)
	Metformin	4 (6.1)
	Sitagliptin	3 (4.5)
	I don’t know	6 (9.1)
Score distribution (*n* = 66)	Frequency(n)	Percentage (%)
0	0	0.0
1	0	0.0
2	0	0.0
3	1	1.5
4	3	4.5
5	10	15.2
6	8	12.1
7	11	16.7
8	12	18.2
9	13	19.7
10	7	10.6
11	1	1.5
Knowledge of Beers’ Criteria		
Poor Knowledge (< 80)	45	68.2
Good Knowledge (> 80)	21	31.8

As shown in Table 6 family medicine physicians’ knowledge of Beers’ criteria was poor, respondents that scored below 80% were (20; 64.5%). Though, (26; 83.8%) of the family medicine physicians knew that metoclopramide should be avoided unless for gastroparesis. However, (30; 96.7%) of the respondents’ had the knowledge that Corticosteroids plus NSAIDs should be avoided because it increases the risk of peptic ulcer disease or Gastro-Intestinal bleeding.

**Table 6:** Family medicine physicians’ knowledge of Beers’ criteria for potentially inappropriate prescribing in elderly (*n* = 31)

Variables	Response Categories	Frequency (%)
Metoclopramide should be avoided unless for gastroparesis with a duration not exceeding 12 weeks except in rare cases because it can cause extrapyramidal side-effects. Correct answer: Yes	Yes	26 (83.9)
	No	2 (6.5)
	I don’t Know	3 (9.7)
Proton-pump Inhibitors should be used for a maximum of 8 weeks in elderly patients except for high-risk patients, erosive oesophagitis, and pathological hypersecretory condition. Correct answer: Yes	Yes	21 (67.7)
	No	3 (9.7)
	I don’t Know	7 (22.6)
Sertraline would increase the risk of delirium. Correct answer: Yes	Yes	14 (45.2)
	No	5 (16.1)
	I don’t Know	12 (38.7)
SSRIs can increase the risk of falls and should be avoided unless safer alternatives are not available. Correct answer: Yes	Yes	15 (48.4)
	No	6 (19.4)
	I don’t Know	10 (32.3)
Benzodiazepines can increase the risk of falls and fractures in elderly patients. Correct answer: Yes	Yes	29 (93.5)
	No	0 (0.0)
	I don’t Know	2 (6.5)
First Generation anti-histamines (Chlorpheniramine) can increase the risk of anticholinergic effects in elderly patients. Correct answer: Yes	Yes	18 (58.1)
	No	7 (22.6)
	I don’t Know	6 (19.4)
Corticosteroids plus NSAIDs should be avoided because it increases the risk of peptic ulcer disease or Gastro-Intestinal bleeding. Correct answer: Yes	Yes	30 (96.8)
	No	0 (0.0)
	I don’t Know	1 (3.2)
Aspirin 325 mg daily may exacerbate existing ulcers or cause new additional ulcers. Correct answer: Yes	Yes	30 (96.8)
	No	0 (0.0)
	I don’t Know	1 (3.2)
Long-acting sulfonylurea (glimepiride, glyburide) should be avoided due to their risk of prolonged hypoglycemia. Correct answer: Yes	Yes	22 (71.0)
	No	8 (25.8)
	I don’t Know	1 (3.2)
A 72-year-old patient has type II diabetes mellitus. The patient is newly diagnosed with hypertension. The patient has experienced recurrent events of hypoglycemia. The patient has no other medical illnesses. Which of the following medications would you consider to AVOID? Correct answer: hydrochlorothiazide.	Metoprolol	11 (35.5)
	Amlodipine	0 (0.0)
	Hydrochlorothiazide	15 (48.4)
	Losartan	1 (3.2)
	I don’t know	4 (12.9)
A 76-year-old patient is newly diagnosed with type II diabetes mellitus. The patient has no other medical illness. You are going to start an oral antidiabetic agent for this patient. Which of the following medications would you consider AVOIDING? Correct answer: Glibenclamide	Rosiglitazone	6 (19.4)
	Glibenclamide	17 (54.8)
	Metformin	1 (3.2)
	Sitagliptin	3 (9.7)
	I don’t know	4 (12.9)
Score distribution (*n* = 31)	Frequency(n)	Percentage (%)
0	0	0.0
1	0	0.0
2	1	3.2
3	0	0.0
4	1	3.2
5	1	3.2
6	6	19.4
7	5	16.1
8	6	19.4
9	4	12.9
10	6	19.4
11	1	3.2
Knowledge of Beers’ Criteria		
Poor Knowledge (< 80)	20	64.5
Good Knowledge (> 80)	11	35.4

### Barriers to respondents use of Beers’ criteria for potentially inappropriate prescribing in elderly

Most hospital pharmacists stated lack of time to counsel each patient (45; 68.2%), excess workload (55; 83.3%), cost of medication, and inability to follow up with patients (49; 74.2%) as a barrier to use of Beers’ Criteria for potentially inappropriate prescribing in elderly. Details in Table 7. Barriers reported by family medicine physicians were lack of time to counsel each patient (17; 54.8%), excess workload (22; 70.9%), and cost of medication (20; 64.6%). See Table 8.

**Table 7:** Barriers to hospital pharmacists’ use of Beers’ criteria for potentially inappropriate dispensing in elderly (*n* = 66)

Variables	Yes *n* (%)	No *n* (%)	I don’t know *n* (%)
Lack of time to counsel each of the patients.	45 (68.2)	14 (21.2)	7 (10.6)
Excess workload.	55 (83.3)	5 (7.6)	6 (9.1)
I hardly come in contact with elderly patients.	10 (15.2)	51 (77.3)	5 (7.6)
Lack of counseling skills.	4 (6.1)	58 (87.9)	4 (6.1)
Lack of knowledge of Beers’ criteria.	25 (37.9)	38 (57.6)	3 (4.5)
Lack of adequate information from the patient.	21 (31.8)	40 (60.6)	5 (7.6)
Impatient Patients.	32 (48.5)	29 (43.9)	5 (7.6)
Inability to follow-up patients.	49 (74.2)	12 (18.2)	5 (7.6)
Lack of acceptable Therapeutic alternatives.	38 (57.6)	20 (30.3)	8 (12.1)
Potential drug-drug interaction.	24 (36.4)	37 (56.1)	5 (7.6)
Cost of medication.	49 (74.2)	10 (15.1)	7 (10.6)
Lack of immediate access to information on the Beers’ criteria when required.	43 (65.2)	16 (24.2)	7 (10.6)
Inconvenient work environment, consultation room.	42 (63.6)	19 (28.8)	5 (7.6)
Communication Barrier.	45 (68.2)	17 (25.8)	4 (6.1)
Lack of financial incentives for additional counseling.	37 (56.1)	22 (33.3)	7 (10.6)

**Table 8:** Barriers to family medicine physicians’ use of Beers’ criteria for potentially inappropriate prescribing in elderly (*n* = 31)

Variables	Yes *n* (%)	No *n* (%)	I don’t Know *n* (%)
Lack of time to counsel each of the patients.	17 (54.8)	12 (38.7)	2 (6.5)
Excess workload.	22 (70.9)	7 (22.6)	2 (6.5)
I hardly come in contact with elderly patients.	1 (3.3)	29 (93.5)	1 (3.2)
Lack of counseling skills.	2 (6.5)	27 (87.1)	2 (6.5)
Lack of knowledge of Beers’ criteria.	10 (32.2)	19 (61.3)	2 (6.5)
Lack of adequate information from the patient.	7 (22.6)	20 (64.5)	4 (12.9)
Impatient Patients.	8 (25.8)	17 (54.8)	6 (19.4)
Inability to follow-up patients.	12 (38.7)	17 (54.8)	2 (6.5)
Lack of acceptable therapeutic alternatives.	10 (32.3)	17 (54.8)	4 (12.9)
Potential drug- drug interaction.	11 (35.4)	15 (48.3)	5 (16.3)
Cost of medication.	20 (64.6)	7 (22.5)	4 (12.9)
Lack of immediate access to information on the Beers’ criteria when required.	15 (48.4)	10 (32.2)	6 (19.4)
Inconvenient work environment, consultation room.	14 (45.1)	15 (48.4)	2 (6.5)
Communication Barrier.	6 (19.4)	22 (70.9)	3 (9.7)
Lack of financial incentives for additional counseling.	8 (25.9)	22 (70.9)	1 (3.2)

### Respondents suggested measures to improve their practice by use of Beers’ criteria for potentially inappropriate prescribing in elderly

All hospital pharmacists (66; 100%) and most family medicine physicians (28; 90.3%) agreed that increasing awareness through workshops and seminars would improve their use of Beers’ criteria. All hospital pharmacists (66; 100%) agreed that interaction between healthcare professionals on how to tackle the issue of inappropriate medication use by elderly patients and having regular clinical meetings about inappropriate medication encountered should be encouraged to improve hospital pharmacists’ practice. Most (29; 93.5%) believed that interaction between healthcare professionals on how to tackle the issue of inappropriate medication use by elderly patients and counseling on adherence and appropriate medication use should be encouraged for elderly patients during health talks. See Table 9.

**Table 9:** Respondents suggested measures to improve their practice using Beers’ criteria for potentially inappropriate prescribing in elderly

Variables	Hospital Pharmacists Frequency (%) (*n* = 66)	Physicians Frequency (%) (*n* = 31)
Increasing awareness of Healthcare Professionals on Beers’ criteria through workshops, seminars		
Yes	66 (100.0)	28 (90.3)
No	0 (0)	3 (9.7)
Interaction between healthcare professionals on how to tackle the issue of inappropriate medication use by elderly patients.		
Yes	66 (100.0)	29 (93.5)
No	0 (0)	2 (6.5)
Having regular clinical meetings about inappropriate medication encountered.		
Yes	63 (95.5)	30 (96.8)
No	3 (4.5)	1 (3.2)
When older patients are being given health talk, the counseling on adherence and appropriate medication use should be encouraged.		
Yes	66 (100.0)	29 (93.5)
No	0 (0)	2 (6.5)

### Factors associated with knowledge (on Beers’ criteria) and practice of hospital pharmacists to prevent potentially inappropriate medication use in elderly patients

The additional qualification possessed by hospital pharmacists had no statistically significant association with their knowledge and practice compared to hospital pharmacists with only a Bachelor of pharmacy degree. The respondents’ years of experience had no statistically significant association with their knowledge and practice. There is also no statistically significant association between hospital pharmacists’ awareness of the criteria for potentially inappropriate prescribing like Beers’ and knowledge of Beers’ criteria (*p* = 0.369). There is no statistically significant association between awareness of Beers’ criteria for potentially inappropriate prescribing and the practice of hospital pharmacists (*p* = 0.303). See Table 10.

**Table 10:** Factors Associated with Knowledge (on Beers’ Criteria) and Practice of Hospital Pharmacists to prevent potentially inappropriate medication use in elderly patients (n = 66)

Variables	Poor Knowledge (%)	Good Knowledge (%)	Poor Practice (%)	Good Practice (%)
**Qualifications**				
Bachelor of Pharmacy	43 (68.3)	20 (31.7)	10 (15.9)	53 (84.1)
Pharm D	2 (66.7)	1 (33.3)	1 (33.3)	2 (66.7)
	[χ^2^ = 0.690]	[*p* = 0.954]	[y = 0.619]	[*p* = 0.431]
**Additional Qualifications**				
Bachelor of Pharmacy	43 (68.3)	20 (31.7)	10 (15.9)	53 (84.1)
Additional qualifications	21 (63.3)	12 (36.4)	3 (9.9)	30 (90.9)
	[χ^2^ = 7.976]	[*p* = 0.240]	[y = 0.254]	[*p* = 0.615]
**Years of Practice**				
Less than one	2 (66.7)	1 (33.3)	0 (0.0)	3 (100)
1-5	20 (76.9)	6 (23.1)	5 (19.2)	21 (80.8)
6-10	13 (68.4)	6 (31.6)	5 (26.3)	14 (73.7)
11-15	7 (53.8)	6 (46.2)	1 (7.7)	12 (92.3)
More than 15 years	3 (60.0)	2 (40.0)	0 (0.0)	5 (100.0)
	[y = 1.539]	[*p* = 0.215]	[y = 1.245]	[*p* = 0.265]
**Heard of criteria for potentially inappropriate prescribing.**				
Yes	18 (75.0)	6 (25.0)	2 (8.3)	22 (91.7)
No	27 (64.3)	15 (35.7)	9 (21.4)	33 (78.6)
	[χ^2^ = 0.808]	[*p* = 0.369]	[y = 1.886]	[*p* = 0.303]
**Number of Patients above the age of 65 years seen in a day.**				
< 10	10 (71.4)	4 (28.6)	3 (24.1)	6 (78.6)
11-20	17 (73.9)	6 (26.1)	3 (13.0)	20 (87.0)
21-30	11 (64.7)	6 (35.3)	2 (11.8)	15 (88.2)
31-40	6 (60.0)	4 (40.0)	2 (20.0)	8 (80.0)
41-50	0 (0.0)	1 (100.0)	0 (0.0)	1 (100.0)
Greater than 50	1 (100.0)	0 (0.0)	1 (100.0)	0 (0.0)
	[y = 3.429]	[*p* = 0.634]	[y = 6.020]	[*p* = 0.304]
**Knowledge of Beers’ Criteria**				
Poor Knowledge (< 80%)			7 (15.6)	38 (84.4)
Good knowledge (> 80%)			4 (19.0)	17 (81.0)
			[y = 0.126;	*p* = 0.733]

χ^2^ **=** Chi-square, significant *p*-value < 0.05, y = linear by linear association

## Discussion

Potentially inappropriate medication prescribing and dispensing by physicians and pharmacists is a common healthcare challenge among the elderly, which can lead to increased healthcare costs, morbidity, and mortality in the elderly. In Nigeria, studies have evidenced a high prevalence of potentially inappropriate prescribing among the elderly using Beers’ criteria and STOPP and START as measuring tools [5–8]. This brings forth the need for healthcare professionals to reduce the occurrence by having adequate knowledge of existing criteria, careful medication review, and patient education.

It is evidenced that pharmacists are an important aspect in improving the appropriateness of medication use among the elderly through medication reviews and interdisciplinary approaches in the care of elderly patients [16]. However, this is the first study in Nigeria assessing the knowledge and practice of healthcare professionals on the use of guidelines for potentially inappropriate medicines and existing criteria among healthcare professionals.

Majority of the physicians in this study had satisfactory practice by considering the appropriate factors when prescribing to their patients indicating a good understanding of therapeutic issues among the elderly. This finding was similar to a previous study carried out in Italy which used clinical vignettes to assess the prescribing of primary care physicians [21]. Findings from this study showed that counseling was carried out among hospital pharmacists which is a significant component of pharmaceutical care [22]. Pharmacists in this study had satisfactory practice in relation to elderly patients by regularly asking questions to assess the appropriateness of medications. This is comparable to findings from previous studies reporting good practices among pharmacists regarding asking appropriate questions, considering age, and referring to other sources of information [10, 18].

Screening tools, such as the Beers’ criteria, STOPP/START, and Medication Appropriateness index criteria, are a group of explicit and implicit classifications of medicines used for the review of elderly patients’ medications. Medications on these lists have been found to contribute significantly to morbidity in elderly patients [23, 24]. Most of the physicians in this study were not aware of any of these criteria. This lack of awareness might be related to the unavailability of guidelines and standard operating procedures to be used while prescribing for the elderly. This finding is similar to a study carried out in Italy among primary care physicians and in the United States among family and internal medicine residents and attending doctors [15, 21].

This study shows family medicine physicians had high self-assessed confidence in appropriate prescribing for elderly patients despite their poor awareness of the screening tools and unsatisfactory knowledge of the existing criteria. This might be because many physicians have undergone one training or the other in the care of the elderly and may use a generalized approach to address clinical scenarios in relation to prescribing issues in adults. This finding correlates with a previous study by Magin et al. [25].

The results of the current study found that many hospital pharmacists had confidence in their care for elderly patients, which differs from a study carried out in Malaysia among hospital and clinical pharmacies reporting only one-third had confidence in their care for elderly patients [10]. This could be because they are more exposed to all aspects of geriatric care due to pharmacy rotations and may have had some training in geriatric pharmacy.

The knowledge score of physicians in this study is below average with most physicians having satisfactory knowledge in therapeutic areas of hypertension, diabetes mellitus, and gastrointestinal tract which can be due to the fact that they are one of the most common diseases in Nigeria. Our study reinforces earlier findings that physician knowledge of criteria for potentially inappropriate prescribing is inadequate and unsatisfactory [15, 21]. The unsatisfactory knowledge of physicians in this study might be contributory to the high prevalence of potentially inappropriate prescribing among the elderly.

The results of the knowledge score of hospital pharmacists showed that only about a third of the participants had satisfactory knowledge. This revealed that most of the participants possessed inadequate knowledge despite the high percentage of elderly patients seen by them every day. Similar results were found in studies conducted in other countries regarding primary care physicians, hospital physicians, and hospital pharmacists [10, 15, 21].

The major barriers to practice listed by the physicians were lack of therapeutical alternatives, potential drug-drug interactions, cost of medication to patients, limited knowledge of potentially inappropriate medications, extra time needed to consult the criteria, poor communication, and lack of formal education of prescribing guidelines. Successful approaches to optimize prescribing have been either educational or administrative. Educational approaches (e.g. one-on-one sessions, academic detailing, and continuing medical education) have been shown to influence decision- making, while administrative approaches attempt to enforce policies to curtail the undesired practice. This is similar to those listed in previous literature [15, 21, 26, 27]. In addition to those listed in the literature, other barriers listed in our study by family medicine physicians are inadequate information on patients and an inconvenient work environment.

Barriers to practice listed by the pharmacists in this study are potential drug-drug interactions, cost of medication to patients, limited knowledge of potentially inappropriate medications, extra time needed to consult the criteria, poor communication, and lack of formal education on prescribing guidelines. These barriers are similar to those listed in literature [15, 21, 26, 27]. It is well established that polypharmacy increases the risk for potentially inappropriate prescribing, adverse drug events, and drug–drug interactions. Although it may sometimes be unavoidable in elderly patients, its negative effects can be reduced by an interdisciplinary review of the patient’s case and medications, discontinuation of unnecessary medications, and the use of explicit criteria for prescribing in geriatrics [6, 10, 14].

Most physicians in our study suggested that having regular clinical meetings about inappropriate medication encountered and interaction between healthcare professionals on how to tackle the issue of inappropriate medication use by elderly patients can be ways to improve the use of the existing criteria for detecting potentially inappropriate prescribing among physicians. This correlates with previous findings which reported that an interdisciplinary approach by healthcare professionals helps reduce medication errors by reducing PIMs use [28–31].

Pharmacists considered continuous professional development courses as one of the best measures to enhance their competency and knowledge of Beers’ criteria for potentially inappropriate prescribing among the elderly. This is consistent with a study carried out in Malaysia among pharmacists and pharmacy students on their perception of geriatric pharmacotherapy education [32].

Overall, the knowledge and awareness of respondents on Beers’ criteria for potentially inappropriate medications was unsatisfactory, signifying the need for the awareness and training of respondents. The knowledge deficit of our respondents may be contributory to the high prevalence of potentially inappropriate medication use in elderly patients among other factors. In addition to improving the knowledge and awareness of healthcare professionals on potentially inappropriate medications, interdisciplinary approach of healthcare professionals can also help reduce potentially inappropriate medication use in the elderly [33].

The strength of this study includes the use of a clinical vignette based on the 2019 Beers Criteria. Research has shown that clinical vignette-based surveys produce better measures of quality of care than medical record reviews when used to assess healthcare professional approaches to diagnosis and treatment [34, 35]. By using this method to determine hospital and physicians’ knowledge, we found about one-third of the healthcare professionals achieved a high score on the clinical vignettes. It would be interesting to validate these findings with a larger sample size. Nonetheless, this study has provided insight into knowledge, practice, confidence, and barriers of hospital pharmacists and physicians on potentially inappropriate medication use among the elderly. It will also contribute valuable baseline evidence to the literature regarding the use of guidelines, such as Beers’ criteria to prevent potentially inappropriate prescribing among the elderly in this setting.

## Limitations

Limitations of our study include reliance on hospital pharmacists’ and physicians’ reports of the use of guideline and barriers to the use of guidelines. Although the Beers criteria can help in identifying poor prescribing practices, convincing evidence to show that they consistently reduce the incidence of adverse drug events, unjustified healthcare resources utilization, or mortality is lacking. The study was also limited to a small sample size and possibly did not have the power to detect a true correlation between the knowledge and practice of these healthcare professionals to prevent potentially inappropriate medication use among the elderly. Our sample may not reflect the general physician and hospital pharmacist population in terms of knowledge and practice settings because only family medicine physicians and pharmacists at the University College Hospital, Ibadan were surveyed.

## Conclusion

The practice of healthcare professionals that participated in this study was satisfactory, but their knowledge of Beers’ criteria was unsatisfactory. Regardless of their knowledge scores, most healthcare professionals are confident with their prescribing and care provided for the elderly. Educational interventions need to be integrated into the training and continuing medical education process to improve medication prescribing for the elderly. Thus, creating awareness among healthcare professionals and training healthcare professionals on Beers’ criteria through different routes like the Mandatory Continuing Professional Development (MCPD), seminars, workshops, etc. can help improve their knowledge. A multidisciplinary approach to reducing potentially inappropriate prescribing would greatly help reduce the incidence of potentially inappropriate medication use among elderly patients.

## Recommendation

In Nigeria, community pharmacies are one of the first points for the purchase of drugs for treating common ailments and the initial source of counsel regarding illness and drug therapy. Pharmacists’ density in Nigeria is still significantly low at less than one pharmacist per 10,000 population compared to density in other countries such as Jordan (20.87), Canada (10.17), USA (8.82), and the United Kingdom (8.08) [36]. In Nigeria, hospital pharmacists see more elderly than community pharmacists, nonetheless, it will be recommended that future studies investigate community pharmacists’ awareness of potentially inappropriate medication, and the various criteria to reduce potentially inappropriate medications use in a bid to reduce the dispensing and subsequent use of these medications.

## Data Availability

The datasets used and/or analysed during the current study are available from the corresponding author on reasonable request.

## Abbreviations

BPharm: Bachelor of Pharmacy
FWACP: Fellow of West African College of Physicians
MBBS: Bachelor of Surgery, Bachelor of Medicine
MPharm: Master of Pharmacy
MPH: Master of Public Health
MWACP: Member, West African College of Physician
NYSC: National Youth Service Corps
Pharm D: Doctor of Pharmacy
PPO: Potential Prescribing Omissions
START/STOPP: Screening tool of older persons’ prescriptions or the screening tools to alert doctors to right treatment
UCH: University College Hospital
WAPCP: West African Postgraduate College of Pharmacist
